# Confidential Enquiry into Maternal Deaths in Namibia, 2018–2019: A Local Approach to Strengthen the Review Process and a Description of Review Findings and Recommendations

**DOI:** 10.1007/s10995-023-03771-9

**Published:** 2023-09-30

**Authors:** Steffie Heemelaar, Beatrix Callard, Hilma Shikwambi, Jana Ellmies, Wilhelmina Kafitha, Jelle Stekelenburg, Thomas van den Akker, Shonag Mackenzie

**Affiliations:** 1 National Maternal Death, Stillbirth and Neonatal Death Review Committee, Division of Quality Assurance, Ministry of Health and Social Services, Windhoek, Namibia; 2 Department of Obstetrics and Gynaecology, Leiden University Medical Center, Leiden, The Netherlands; 3 Department of Nursing and Midwifery, International University of Management, Windhoek, Namibia; 4 Independent Midwives Association of Namibia, Windhoek, Namibia; 5 Division of Quality Assurance, Ministry of Health and Social Services, Windhoek, Namibia; 6 Department of Health Science, Global Health, University Medical Center Groningen, Groningen, The Netherlands; 7 Department of Obstetrics and Gynaecology, Medical Center Leeuwarden, Leeuwarden, The Netherlands; 8 Athena Institute, VU University, Amsterdam, The Netherlands; 9 Department of Obstetrics and Gynaecology, University of Namibia, Windhoek, Namibia

**Keywords:** Namibia, Maternal mortality, HIV

## Abstract

**Objectives:**

First objective was to strengthen the national maternal death review, by addressing local challenges with each step of the review cycle. Second objective was to describe review findings and compare these with available findings of previous reviews.

**Methods:**

Confidential Enquiry into Maternal Deaths methodology was used to review maternal deaths. To improve reporting, the national committee focussed on addressing fear of blame among healthcare providers. Second focus was on dissemination of findings and acting on recommendations forthcoming the review. Reviewed were reported maternal deaths, that occurred between 1 April 2018 and 31 March 2019.

**Results:**

Seventy maternal deaths were reported; for 69 (98.6%) medical records were available, compared to 80/119 (67.2%) in 2012–2015. Reported maternal mortality ratio increased with 48% (92/100,000 live births compared to 62/100,000 in 2012–2015). Obstetric haemorrhage was leading cause of death in the past three reviews. The “no name, no blame” policy, aiming to identify health system failures, rather than mistakes of individuals, was repeatedly explained to healthcare providers during facility visits. Recommendations based on findings of the review, such as retaining experienced staff, continuous in-service training and guidance, were shared with decision makers at regional and national levels. Healthcare providers received training based on review findings, which resulted in improved management of similar cases.

**Conclusions for Practice:**

Enhanced implementation of Confidential Enquiry into Maternal Deaths was possible after addressing local challenges. Focussing on obtaining trust of healthcare providers and feeding back findings, resulted in better reporting and prevention of potential maternal deaths.

**Supplementary Information:**

The online version contains supplementary material available at 10.1007/s10995-023-03771-9.

## Significance

*What is already known on this subject?* To analyse and improve quality of care, a national maternal death review is recommended. In Namibia successful implementation was hampered by a blame culture. Around the world, healthcare providers are frequently blamed by decision makers when a woman dies. Limited literature is available on how this issue could be addressed.

*What this study adds?* It was possible to improve implementation by focussing on increasing trust of healthcare providers in the review process. This was achieved by addressing fear of being blamed, dissemination of findings and acting on the recommendations forthcoming from the review.

**Supplementary Information:**

The online version contains supplementary material available at 10.1007/s10995-023-03771-9.

## Introduction

Namibia has a high maternal mortality ratio (MMR) of an estimated 215/100,000 live births in 2020 (World Health Organization, 2023). This is three times higher than the average reported MMR for upper-middle-income countries (USAID, 2021). The Namibian government is committed to reducing this MMR and reaching the target of 70/100,000 livebirths in 2030, as set by the sustainable development goals (SDG3) (Geingob, 2016). Namibia is one of the least densely populated countries in the world with 2.8 people per square kilometre (Namibia Statistics Agency, 2017). Key indicators suggest high access to maternity care, with 96.6% of women having at least one antenatal visit and 87.4% giving birth in a health facility in 2013 (Ministry of Health and Social Services, 2014). However, these figures do not indicate whether women reached the facility in time, nor do they provide information about quality of provided care.

**Fig. 1:**
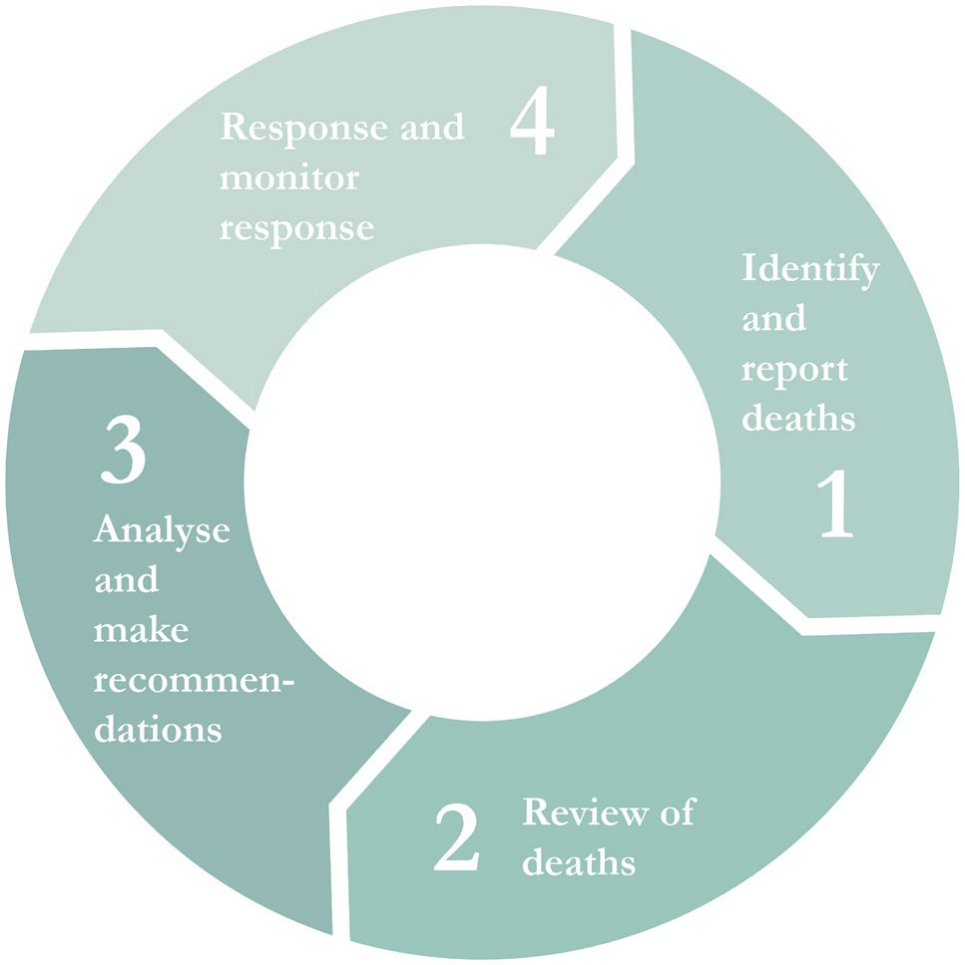
Confidential enquiry into maternal deaths audit cycle

To assess the underlying causes and address the high MMR, a national maternal death review committee has analysed maternal deaths since 2010, using the Confidential Enquiry into Maternal Deaths (CEMD) methodology (Lihongeni and Indongo, 2012; World Health Organization, 2013). This methodology is based on a continuous audit cycle as illustrated in Fig. 1. CEMD can provide more insights into mortality rates, causes of deaths and modifiable factors contributing towards these deaths. Subsequently recommendations need to be implemented, based on these findings, in order to improve maternal outcome.

In Namibia the CEMD was not functioning well due to several challenges. Lack of trust in CEMD by healthcare providers resulted in severe underreporting and medical notes not being submitted for the review (step 1). In the review period 2012–2015, fear of being blamed for a woman’s death, no feedback of review findings and no acting on the recommendations forthcoming the review, were common expressed concerns by healthcare providers. Furthermore, due to lack of staff within the national committee, a minority of deaths that occurred between 2015 and 2018 were reviewed (step 2) and the report was not completed for the review of 2012–2015 (step 3). As a result of the challenges with the first three steps, very few recommendations forthcoming the review were implemented (step 4).

In 2018 several additional members joined the national committee. For the review period of 1st of April 2018 up to the 31st of March 2019 this new national committee focused on improved effectiveness of the CEMD audit cycle, by addressing fear of being blamed, dissemination of findings at all levels and acting on the recommendations forthcoming from the enquiry. This paper aims to describe this approach and results of the enhanced implementation of CEMD in Namibia. Secondly, we describe the findings of the review for 2018–2019 and compare these with available findings of previous reviews.

## Methods

### CEMD Process

The division of Quality Assurance of the Ministry of Health and Social Services (MoHSS) is responsible for the CEMD process. The CEMD process is described in detail in the national guidelines and summarized in this paragraph (Ministry of Health & Social Services, 2019b). It is a legal requirement to notify every maternal death to MoHSS (Ministry of Health & Social Services, 2019b). Maternal deaths (MD) are initially reviewed at facility level by the midwifery and medical staff working in obstetrics. From facility level, matrons report to the regional CEMD committee. At regional level, all documents are anonymized by administrative staff prior to review. All deaths are reviewed, and a summary report is written for each mortality meeting.

At national level, all deaths are reviewed by an expert committee. Members of this committee are appointed by the MoHSS and do not receive any remuneration as improving quality of care is seen as one of the responsibilities of all staff. A single underlying cause of death is identified, defined as the cause which initiated the chain of events leading to death. From 2018 causes have been defined according to the ICD-MM definitions to allow international comparison (World Health Organization, 2012). Through audit of medical records, quality of provided care is assessed and identified modifiable factors are categorized into either patient, administrative (e.g. lack of equipment, medical supplies, transport or supervision) or healthcare provider related. In conclusion, the provided care is assessed as either ‘good care’, ‘improvements to care would not have prevented the death’, or ‘improvements to care which may have prevented the death’.

For each maternal death review findings are captured on a structured form (Online Appendix I), together with background data extracted from medical records. After reviewing all reported deaths that occurred within a financial year, a meeting is set up to write the annual report summarizing key drivers of maternal mortality and recommendations on how these can be addressed.

### Interventions to Improve Effectiveness of the CEMD Audit Cycle

The national committee has approximately 15 to 20 appointed members. In 2018 several members left the committee, who had not been able to commit for the past reviews. Several new members, who had contributed substantially to reviews at facility or regional level, were appointed to the national committee. For the 2018–2019 review, the national committee consisted of four obstetricians working in both the national referral hospital, the medical school and/or a private hospital, a medical doctor working in an Obstetrics and Gynaecology department in a public hospital, senior nurses and midwives from both public and private facilities, representatives of the Independent Midwives Association of Namibia and senior lecturers of all nursing-midwifery training institutions. A quorate meeting was when at least two medical doctors of which one was an obstetrician and at least two nurse/midwives were present.

To increase trust of healthcare providers in the review process, the committee focussed on: addressing fear of being blamed, dissemination of findings at all levels and acting on the recommendations forthcoming from the enquiry. Whenever a committee member visited a facility, e.g. for data collection, provision of feedback or other quality improvement projects, the anonymous and confidential aspect of the review was explained. The importance of CEMD as a tool to address system failures, rather than individual mistakes, was emphasized. Lastly, the most recent CEMD guidelines were discussed including a paragraph stating that the findings of CEMD could not be used for any medico-legal or disciplinary procedure and, if needed, MoHSS would provide legal support to ensure this (Ministry of Health & Social Services, 2019b). During these visits working conditions were noted, as well as staffing, hospital facilities and equipment.

### Data Collection and Analysis

MD was defined as death of a woman while pregnant or within 42 days of termination of pregnancy or birth, from any cause related to or aggravated by the pregnancy or its management, but not from accidental or incidental causes. All MD were included that had occurred between 1st of April 2018 and 31st of March 2019 and had been reported to the national CEMD committee. Data collection is described in the section ‘CEMD process’. Data analysis consisted of frequencies of clinical variables and review findings. To assess trends over time, findings were compared with available data of the review of 2008–2012, of which the report was published, and the review of 2012–2015, of which the report was not completed (Lihongeni & Indongo, 2012).

## Results

### Reporting

There were 76,498 live births and 70 maternal deaths reported between 1 April 2018 and 31 March 2019 giving an MMR of 92/100,000 live births (LB). This is a rise of 48% compared with the reported MMR in 2012–2015 (62/100,000 LB). For 69/70 (98.6%) deaths medical records and review forms, completed at facility level, were available and reviewed by the national committee, compared to 80/119 (67.2%) reviewed in 2012–2015. The 2018–2019 review included eight MD which occurred at home, while no ‘home deaths’ were reported in the previous reviews. For the review of 2008–2012 154 deaths were reviewed, but MMR could not be calculated as total live births was not available for that period (Lihongeni & Indongo, 2012). Furthermore, it was unclear how many deaths remained unreviewed due to missing medical records.

### Review Findings

Table 1 presents baseline characteristics of reviewed deaths, compared to available data from previous reviews. Most women attended ANC, 49/70 (70.0%), which was similar to 62/80 (77.5%) MD of the 2012–2015 review. Table 2 summarizes the causes of MD. In 2018–2019, most maternal deaths were from direct causes, 35/70 (50%), compared to 28/70 (40%) deaths from indirect causes and this was similar in the previous reviews. Obstetric haemorrhage was the leading cause of death in all three reviews. In 2018–2019 hepatitis E was also one of the leading causes of death, while there were no MD due to hepatitis E in previous reviews. In the review of 2008–2012 and 2012–2015 ‘HIV/AIDS related’ was one the leading causes of death. In 2018–2019 22/70 (31.4%) women were HIV-positive, of whom seven died had an AIDS-defining condition. Five of these seven women died due to tuberculosis. None of the HIV-positive women were offered isoniazid therapy, to prevent tuberculosis co-infection, as recommended in the Namibian HIV guidelines (Ministry of Health & Social Services, 2016).

**Table 1:** Characteristics of all maternal deaths

	2018–2019		2012–2015		2008–2012	
	(N = 70)	%	(N = 80)	%	(N = 154)	%
**Age**						
< 20	5	7.1	5	6.3	N/A	N/A
20–34	48	68.6	46	57.5	N/A	N/A
≥ 35	16	22.9	28	35.0	N/A	N/A
Unknown	1	1.4	1	1.3	N/A	N/A
**Parity**						
Para 0	10	14.3	N/A	N/A	N/A	N/A
Para 1–3	42	60.0	N/A	N/A	N/A	N/A
≥ 4	13	18.6	N/A	N/A	N/A	N/A
Unknown	5	7.1	N/A	N/A	N/A	N/A
**ANC attendance**						
Yes	49	70.0	62	77.5	N/A	N/A
No ANC	12	17.1	12	15.0	N/A	N/A
Not applicable, pregnancy < 20 weeks	4	5.7	N/A	N/A	N/A	N/A
Unknown	5	7.1	6	7.5	N/A	N/A
**HIV status**						
Positive	22	31.4	17	21.3	N/A	N/A
Negative	38	54.3	16	20.0	N/A	N/A
Unknown	10	14.3	47	58.8	N/A	N/A
**Mode of birth**	(N = 50)^a^			(N = 96)^a^		
Normal vaginal birth	31	62.0	N/A	N/A	67	69.8
Instrumental birth	1	2.0	N/A	N/A	0	0.0
Caesarean section	17	34.0	N/A	N/A	29	30.2
Laparotomy uterine rupture	1	2.0	N/A	N/A	0	0.0
**Facility for birth/miscarriage**						
Home	8	11.4	N/A	N/A	17	17.7
Health centre	2	2.9	N/A	N/A	N/A	N/A
Hospital	45	64.3	N/A	N/A	N/A	N/A
Unknown	2	2.9	N/A	N/A	N/A	N/A
Pregnant at time of death	13	18.6	N/A	N/A	N/A	N/A

*ANC* antenatal care

^a^Percentages given MD excluding those with a miscarriage or who died antepartum

**Table 2:** Causes of maternal deaths

Causes of maternal death	2018–2019		2012–2015		2008–2012	
	(N = 70)	%	(N = 80)	%	(N = 154)	%
*Direct deaths*	35	50.0	46	57.5	90	58.4
Obstetric haemorrhage	11	15.7	17	21.3	34	22.1
Hypertensive disorder	9	12.9	15	18.8	22	14.3
Pregnancy with abortive outcome	6	8.6	5	6.3	5	3.2
Pregnancy related infection	5	7.1	7	8.8	21	13.6
Other obstetric complications	2	2.9	0	0.0	6	3.9
Anaesthetic death	2	2.9	2	2.5	N/A	N/A
*Indirect deaths*	28	40.0	31	38.8	64	41.6
HIV/AIDS related	*XX*^*a*^		17	21.3	29	18.8
Hepatitis E	11	15.7	0	0.0	0	0.0
Tuberculosis	7	10.0	5	6.3	11	7.1
Medical, not specified	0	0.0	3	3.8	N/A	N/A
Cardiac disease	7	10.0	3	3.8	N/A	N/A
Pneumonia	1	1.4	0	0.0	15	9.7
Other	4	2.9	3	3.8	N/A	N/A
Unknown cause of death	7	10.0	3	3.8	N/A	N/A

*N/A* not available

^a^7 women had AIDS defining conditions, but ICD MM codes were used for cause of death: 5 women died of tuberculosis, 1 due to septic miscarriage and 1 pneumonia

In 2018–2019 most modifiable factors were related to healthcare providers and administrative factors (Table 3). Most delays occurred after a woman had arrived to a health facility. The commonest modifiable factors were ‘lack of expertise, training or education’ (62.3%), ‘problems with recognition and/or diagnosis’ (58.0%), ‘delay in referring the patient’ (55.1%) and ‘delay in initiating critical care due to overburdened facility’ (53.6%). An example for "problems with recognition" is that staff did not recognize signs of hypovolaemic or septic shock. Furthermore, critically ill women could not be transferred to ICU due to a lack of beds (overburdened facility). It was noted that there was lack of access to basic but essential services such as emergency blood or magnesium sulphate in 22 cases. Patient related factors were identified in a few cases, of which delay in seeking care was most common (20/69, 29.0%). Eight women had defaulted their tuberculosis or antiretroviral treatment for HIV. The committee concluded that in 40 (57.1%) of cases, MD may have been prevented if improved care had been provided, Table 3. For three deaths the committee could not determine whether the death was preventable as medical records were incomplete (2) or no records were available (1). Data on modifiable factors was not available for the previous reviews.

**Table 3:** Modifiable factors classified according to patient, health system and healthcare provider related and conclusion national committee

	(N = 69)	%
*Patient related factors*		
No antenatal care	16	23.2
Infrequent antenatal care	5	7.2
Delay in woman seeking care	20	29.0
Refusal of treatment or admission	8	11.6
Unsafe abortion	4	5.8
*Health system related factors*		
Lack of transport from home to health care facility	2	2.9
Lack of transport between health care facilities	9	13.0
Lack of accessibility	3	4.3
Delay initiating critical care (overburdened facility)	37	53.6
Communication breakdown between healthcare providers	13	18.8
Lack of facilities, equipment or consumables	22	31.9
Lack of human resources (doctors/nurses)	29	42.0
Lack of expertise, training or education	43	62.3
Lack of specialist	8	11.6
*Healthcare provider related factor*		
Problem with recognition/diagnosis	40	58.0
Delay in referring patient	38	55.1
Managed at inappropriate level	34	49.3
Incorrect management (incorrect diagnosis)	30	43.5
Sub-standard management (correct diagnosis)	33	47.8
Not monitored/infrequently monitored	30	43.5
Prolonged abnormal monitoring with no action taken	33	47.8

	(N = 70)	%
*Conclusion substandard care*		
Yes, it was a preventable death, improvements to care may have made a difference to outcome	40	57.1
Substandard care, but improvements to care would have made no difference to outcome	17	24.3
No, good care	10	14.3
Unknown, lack of information	3	4.3

For 69/70 maternal deaths, the file was available for assessment of modifiable factors by the national committee

During the review period members of the national committee visited nearly all hospitals at least once. These visits provided useful information for the establishment of key findings and recommendations, in addition to review of medical records only. Common challenges such as shortages of staff or equipment were often not mentioned in a woman’s file as staff consider this the normal situation at the facility. For example, one district hospital had one blood pressure machine, which was shared between several wards. This hospital recorded one MD, where lack of blood pressure monitoring was identified as a modifiable factor.

In the annual report most key findings and recommendations were similar to those of previous reviews, and mainly related to healthcare providers and administrative factors. An important intervention that had been implemented in 2015 to improve quality of care, was the provision of Emergency Obstetric Care training to numerous doctors and nurses, including an instructor course to facilitate continuous training. However, during facility visits in the current review it appeared the continued provision of training was compromised by rotation of trained staff to other departments.

### Feedback of Findings and Response

To improve implementation of recommendations, after the review of 2018–2019, the national committee focused on providing feedback regarding findings and recommendations to all relevant stakeholders and implemented several recommendations themselves.

All recommendations were shared with decision makers at Ministry level in a meeting with all relevant divisions present. During this meeting several issues, such as lack of experienced staff, continuous in-service training and guidance and the availability of essential medication and equipment were discussed. The MoHSS human resources department took immediate steps to retain experienced staff in obstetric departments, and those trained to provide the Emergency Obstetric Care course.

Management staff and healthcare providers were visited in all 14 regions to discuss that regions’ specific cases as well as to feedback important lessons learned at national level, and to follow up on implementation of previous recommendations. For healthcare providers, feedback was provided through a video conference and a two-day conference, which was attended by over 200 doctors and nurses, representing nearly all hospitals. Lastly, instantaneous feedback was provided throughout the review, meaning that if a review at national level identified findings of educational value, these findings were shared in a blame-free manner with healthcare providers at the facility where the death had occurred. For example, after review of a maternal death related to failed intubation, in-service anaesthetic refresher training was provided. A similar event occurred 1 week later in the same facility and both the woman and her baby were saved.

Representatives of the medical and nursing/midwifery training institutions were tasked with the incorporation of adequate training in the identification and management of the commonest conditions contributing to maternal deaths into the respective nursing and medical curricula to prepare future staff appropriately.

Initially reporting had seemed to improve. However, during facility visits it appeared that several deaths had not been reported to national level. Cases were expected to be reported after completion of the review at facility level. Reporting documents should include notes of an audit meeting and autopsy report. Due to various reasons, including overburdened staff or missing documentation, the MD review was not always completed and, therefore, MD not reported. To address this, a brief rapid notification form was introduced, whereby the attending healthcare provider is expected to report the death within 24 h to national level, even if very scarce details pertaining to the death are available at that time of reporting.

Lastly, in order to increase emphasis on the achievements of healthcare providers, the national committee conducted a 6-month ‘Maternal Near-Miss’ surveillance; a nationwide registry collecting quantitative data regarding severe maternal morbidity. This focussed on and acknowledged the number of women whose lives had indeed been saved (Heemelaar et al., 2020).

## Discussion

The national committee aimed to further enhance implementation of the CEMD in Namibia, by increasing trust of healthcare providers in the review process, which appears to have resulted in improved reporting and availability of nearly all medical records. The review findings of 2018–2019 indicate that half of MDs were from direct causes and nearly half from indirect causes. Additionally, ‘in-facility’ delays largely contributed to these deaths.

### Enhanced Implementation of CEMD

To address underreporting the culture of blame needed to be addressed. Fear of being blamed is exacerbated by ‘sensationalist’ media reports, accusing healthcare providers of bad attitudes, providing substandard care and even holding them directly responsible for the death of a woman or baby (Kangootui, 2014; Kooper, 2016; Ministry of Health and Social Services, 2014; Shinana, 2015). Namibia is not alone. Several other countries reported similar counterproductive effects of a blame culture on the performance of confidential enquires (Hodorogea & Friptu, 2014; Lewis, 2014; Melberg et al., 2019; Rousseva et al., 2020; Smith et al., 2017). Unfortunately, across the world, healthcare providers are frequently blamed by leaders or decision makers when a woman dies (Hodorogea & Friptu, 2014; Lewis, 2014; Melberg et al., 2019). When a healthcare provider does make a mistake, the challenging working conditions and failures of the health system are often not taken into consideration, nor addressed in order to prevent similar incidents in the future (Lewis, 2014; Rousseva et al., 2020). The improved reporting in this review period shows that focus on increasing trust of healthcare providers in the review process may be a possible effective strategy to address the blame culture.

Several recommendations forthcoming from the review were similar to those of the previous reviews suggesting a slow response, which is a challenge in many countries and commonly referred to as the ‘knowledge-act gap’ (Jayakody & Knight, 2020; Moodley et al., 2014). The new committee sought to overcome the ‘knowledge-act gap’ through feedback of findings to all relevant stakeholders. For instance, members of the national committee immediately addressed important findings after a review of a maternal death with the wider obstetric and midwifery workforce. Using such timely feedback to facilities, further potential deaths were avoided in the short term. Furthermore, as review findings were combined with observations from facility visits, more feasible and applicable recommendations were drafted and several issues were addressed directly with policy makers.

### Review Findings

The ‘in-facility’ delays were the result of a combination of healthcare providers being understaffed, having insufficient knowledge and skills to timely diagnose and manage complications, and an insufficient supply of basic and essential resources. This combination of challenges is seen in most countries with a high MMR and commonly referred to as ‘Too little, too late (Miller et al., 2016). That most women who died were able to reach health facilities, but that mainly in-facility’ delays contributed to these deaths, suggests that Namibia is now in stage III within the five-step ‘obstetric transition’ as described by the World Health Organization (Souza et al., 2014). This model is based on a common pathway that has been identified among countries that were able to progress from high to low maternal mortality ratios. In stage III women are reaching the health facility, the impact of indirect causes starts to increase and improving quality of maternity care becomes the critical step to achieve a reduction of maternal mortality.

Due to a nationwide outbreak in Namibia in December 2017, hepatitis E was the most common indirect cause. Hepatitis E during pregnancy has an estimated case fatality rate of 20% and outbreaks are mainly seen in Africa and Asia in regions with poor sanitation (Jin et al., 2016). Hepatitis E was not a cause of MD in the previous reviews (Ministry of Health and Social Services, 2019c).

HIV and tuberculosis contributed significantly to maternal mortality in all three reviews, even though treatment coverage rates have significantly improved in the past few years. Antiretroviral treatment was introduced only two decades ago, yet 97.1% of HIV infected females were on antiretroviral treatment in 2017, and of those 92.2% were virally suppressed (Ministry of Health & Social Services, 2019a). Treatment success rate for tuberculosis was estimated at 86% (World Health Organization, 2019). Namibia has one of the highest tuberculosis/HIV coinfection rates in the world, estimated at 35% in 2018 (World Health Organization, 2019). The poor integration of these two services is of great concern as none of the HIV positive pregnant women were offered isoniazid preventive therapy. HIV and tuberculosis were one of the leading causes of death in all three reviews. However, a trend could not be assessed due to the introduction of ICD-MM classification with the last review (Lihongeni & Indongo, 2012).

The high proportion of preventable deaths, challenges with reporting and failed implementation of recommendations from previous reviews were discouraging findings. But looking at other countries, these experiences are not unique. South Africa, which started with CEMD in 1998, had similar recommendations until 2007 and only started to see a reduction in maternal deaths from 2011 (Moodley et al., 2014). In 2014, 57% of the reviewed deaths in South Africa were classified as preventable (Moodley, 2015). But also countries with a low MMR, such as the United Kingdom or the Netherlands identified substandard care in more than half of their reviewed deaths (Knight et al., 2019; Schutte et al., 2010).

### Remaining Challenges

To monitor the impact of implemented interventions, a reliable MMR is essential. Despite improved reporting, the identified MMR in the current review is still far lower than estimated by the demographic health survey or WHO, suggesting underreporting is still present (Maternal Mortality Estimation Inter-Agency Group, 2017; Ministry of Health and Social Services, 2014). Half of the reported ‘out of facility’ deaths occurred within the same region whereas other regions with large rural populations reported no ‘out of facility’ deaths. A survey performed in the five southern regions identified an underreporting of more than 70 percent through national reporting in 2010–2012 (Ministry of Health and Social Services, 2014). The identified rise in the current review may suggest better reporting. However, there may have been an actual rise due to the nationwide hepatitis E outbreak. Large discrepancies between MMRs based on national reporting and vital statistics, as identified in Namibia, were also found in other low and- middle-income countries such as South Africa, Ethiopia and Malawi (Mataya et al., 2014; Melberg et al., 2019; Moodley et al., 2014). Enhanced implementation of CEMD will enable Namibia to produce a more accurate MMR. In the United Kingdom, France and the Netherlands, countries with a low MMR and well-functioning surveillance system, CEMD is the most accurate method for maternal mortality surveillance in comparison to vital statistics (Creanga, 2017).

Secondly, all data pertaining to MD had to be entered manually in an electronic database at national level. This was done by administrative staff, who were supported by several committee members. Such a system is difficult to sustain. Possibilities for electronic data collection at facility or regional level are currently being explored.

Thirdly, the committee has not yet addressed the media and their negative reporting on healthcare providers. During the meeting at ministry level, it was attempted to increase recognition of achievements of healthcare providers by politicians and high government officials. Recognition by supervisors and higher-level officials is important for several reasons. It has a positive effect on the performance of CEMD and the provided quality of care (Lewis, 2014; Rousseva et al., 2020). Furthermore, a study performed in Namibia indicated that lack of recognition was associated with the provision of disrespectful maternity care (Wesson et al., 2018).

To address the in-facility delays, key recommendations included employment of more staff, the provision of continuous postgraduate training and retaining experienced staff. Due to lack of resources this could yet not be implemented. The MoHSS has an active programme of training Namibian specialists and it is anticipated that the number of Specialist obstetricians employed in public hospitals will increase over the next few years.

## Conclusion

CEMD is developed to assess and improve quality of care, but successful implementation is key. In Namibia several challenges with implementation were overcome by focussing on gaining trust of healthcare providers and the provision of guidance and support. We recommend other countries with challenges implementing CEMD effectively, to first identify issues that affect implementation in their setting, as this will allow a locally applicable approach to address these. Subsequently this valuable tool can be used for what it is designed for; to improve maternal outcome.

### Supplementary Information

Below is the link to the electronic supplementary material.

Supplementary file1 (PDF 275 kb)

## Data Availability

All available data are presented in the manuscript and tables.
