# Supplementary material for: Confidential Enquiry into Maternal Deaths in Namibia, 2018–2019: A Local Approach to Strengthen the Review Process and a Description of Review Findings and Recommendations
Source: Matern Child Health J. 2023 Sep 30;27(12):2165–74. doi: 10.1007/s10995-023-03771-9 (PMC10618300; doi:10.1007/s10995-023-03771-9)
Supplement: Supplementary file 1 — Supplementary file1 (PDF 275 kb) [file 10995_2023_3771_MOESM1_ESM.pdf]

Republic of Namibia
Ministry of Health and Social Services

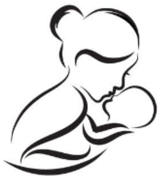

CONFIDENTIAL MATERNAL DEATH REVIEW FORM

NOTE:

1. This form must be completed for all maternal deaths, including deaths from abortion and ectopic gestation related deaths in pregnant women or within 42 days after termination of pregnancy irrespective of duration or site of pregnancy.
2. Circle number where applicable.
3. To be completed by a Medical Practitioner or Registered or Enrolled Nurse/Midwife or the in charge of the unit where the death occurred.
4. Complete the original form. The original remains at the facility where death occurred and two copies must be sent simultaneously to the Regional and National Maternal, Stillbirth and Neonatal Death Review Committee Secretariats *within 7 days of the review*.
5. Attach a copy of the maternal record (ANC or health passport and hospital records). In the copy of the case record, patient's names and all names of health care workers should be crossed out with a black marker to enable a confidential review of the case.
6. Fill in all information that is available. Write N/A if not available or not applicable.
7. Woman's name should not be mentioned but a unique identifier should be given:

   1-2 Abbreviation of region, (e.g. KU, ER, OS, HA etc.)
   3-4 Month of death (e.g. 05, 12 etc.)
   5-6 Year of death (e.g. 21, 19, etc.)
   7-8 Initials of patient or woman in case of stillborn or neonatal death (e.g. AM, SS, TE etc.)
   9-10 Year of birth patient or woman in case of stillborn or neonatal death (e.g. 89, 91, 07 etc.)

   For example:
   Ms Anna Petrus, DOB 23-05-1987, died on 4 April 2017 in Kunene region will be
   KU-04-17-AP-87

8. Contact details for National Level:
   Division Quality Assurance:

   email: ____________________ Fax ____________________

REPUBLIC OF NAMIBIA

MINISTRY OF HEALTH AND SOCIAL SERVICES

CONFIDENTIAL MATERNAL DEATH REVIEW FORM

Woman's name should not be mentioned but a unique identifier should be given:

Unique identifier:

Example: *Anna Petrus, DOB 23-05-1987, died on 4 April 2017 in Kunene will be KU 04 17 AP 87*

		-			-			-			-		
Reporting Region			Month of death			Year of death			Initials of Woman			Woman's year of birth	

1) *Reporting Facility Name:* ____

2) *Region of reporting Facility:*

1	Erongo (ER)	6	Khomas (KH)	11	Oshana (OS)
2	Hardap (HA)	7	Kunene (KU)	12	Oshikoto (OK)
3	Karas (KA)	8	Ohangwena (OH)	13	Otjozondjupa (OT)
4	Kavango East (KE)	9	Omaheke (OM)	14	Zambezi (ZA)
5	Kavango West (KW)	10	Omusati (OU)	99	Unknown (UN)

A. Maternal Demographic details

3) *Woman's Initials:* ___ - ___

4) *Date of Birth:* ______ /_____ / ________ (dd/ mm /yyyy)

5) *Age:* _____

6) *Marital Status:* 1 Married 2 Single 99 Unknown

7) *Nationality:* 1 Namibian 2 Non-Namibian 99 Unknown

8) *Employment:* 1 Employed (incl. self-employed) 2 Unemployed 99 Unknown

9) *Admission to facility:*

1. Date ______ /_____ / ________ (dd/ mm /yyyy) 99 Unknown
2. Time ______ h_____ 99 Unknown

10) *Status on admission:* 1 Stable 2 Critically ill 3 Dead on arrival

11) *Reason for referral:* ____

12) *Referred from:*

1	Self referred (home)	4	District Hospital	7	Private facility
2	Health Clinic	5	Intermediate Hospital		
3	Health Centre	6	National Hospital	99	Unknown

13) *Name of Referring facility:* ____

14) *Region of Referring facility:*

1	Erongo (ER)	6	Khomas (KH)	11	Oshana (OS)
2	Hardap (HA)	7	Kunene (KU)	12	Oshikoto (OK)
3	Karas (KA)	8	Ohangwena (OH)	13	Otjozondjupa (OT)
4	Kavango East (KE)	9	Omaheke (OM)	14	Zambezi (ZA)
5	Kavango West (KW)	10	Omusati (OU)	99	Unknown (UN)

B. Maternal Medical History

15) *Woman's Body Parameters:*
- 1 Height ________cm
- 2 Last Weight ________kg
- 3 MUAC ________cm
- 99 Unknown

16) *Last haemoglobin prior to admission:* 1 ______________g/dl 99 Unknown

17) *Past Medical History:*

1	Hypertension	6	GIT Disorder	11	Epilepsy
2	Cardiac Disease	7	Diabetes Mellitus	12	Mental Health Disorder
3	Respiratory Disease (Chronic)	8	Anaemia	13	Others (Specify) ________________________
4	Renal Disease	9	DVT / PE		
5	Liver Disease	10	TB	14	None
				99	Unknown

18) *Past Surgical or Trauma History:*

1	Myomectomy	4	3rd / 4th degree Tear	7	Poly trauma
2	Salpingectomy	5	Laparotomy (not C/S)	8	Others (Specify) ________________________
3	ERPOC / MVA	6	Pelvic Injury	9	None
				99	Unknown

C. Obstetric (Antenatal) details

19) *Gravidity:* _______ [at start of current pregnancy – all pregnancies including previous abortions]

20) *Parity:* _______ [number of babies delivered > 28weeks or >1000g (dead or alive). excl current pregnancy]

21) *Number of live children:* _______

22) *Number of previous pregnancy losses (<13 weeks):* _______ [at start of current pregnancy]

23) *Number of previous pregnancy losses (≥13 - <28 weeks):* _______ [at start of current pregnancy]

24) *Number of previous Stillbirths (≥28 weeks):* _______ [at start of current pregnancy]

25) *Number of previous Caesarean Sections:* _______ [at start of current pregnancy]

26) *Did woman attend Antenatal Care:* 1 Yes* 2 No 99 Unknown

27) **If yes, how many contacts:* _______

28) *Timing of 1st contact* 1 1st Trimester (<13wks) 2 2nd Trim. (≥13 – 27wks) 3 3rd Trim. (≥28wks) 99 Unknown

29) *Special Investigations:*

=	1	Negative	2	Positive			99	Unknown
Antibodies present	3	No	4	Yes			99	Unknown
Hep B	1	Negative	2	Positive	3	Declined Test	99	Unknown
RPR	1	Negative	2	Positive	3	Declined Test	99	Unknown
Treatment given	4	No	5	Yes	6	Declined Treatment	99	Unknown
HIV	1	Negative	2	Positive	3	Declined Test	99	Unknown
Treatment given	4	No*	5	Yes**			99	Unknown

30) **If NOT on ARVS:* 1. Not yet started 2. Declined ARVs 3. Defaulted 99. Unknown

31) ***If HIV Positive: Treatment started when?*
1 Pre pregnancy 2 This pregnancy at ______ weeks 99 Unknown

32) *If HIV Positive: Latest Test:*
1 Latest CD4 Count #_________ ________ /______ / ________ (dd/ mm /yyyy) 99 Unknown
2 Latest Viral Load #__________ ________ /______ / ________ (dd/ mm /yyyy) 99 Unknown

33) *TB Preventative therapy (TPT) given:*
1 Yes _________ Started ________ /______ / ________ (dd/ mm /yyyy) 4 Defaulted
2 No 3 Declined Treatment 99 Unknown

D. Obstetric details (delivery, termination, miscarriage)

34) *Place of delivery, miscarriage or termination*:*

1 Home (incl BBA)	5 Intermediate Hospital	66 Undelivered
2 Health Clinic	6 National Hospital	88 Transfer between facilities
3 Health Centre	7 Private facility	99 Unknown
4 District Hospital		

** If miscarried, terminated or undelivered – skip to Question 42*

35) *Delivery details:* (if more than triplets, please add details at bottom of page)

1	First Baby	______ /______ / _________ (dd/ mm /yyyy)	99 Unknown
	Time	______ h______	99 Unknown
2	Twin 2	______ /______ / _________ (dd/ mm /yyyy)	99 Unknown
	Time	______ h______	99 Unknown
3	Triplet 3	______ /______ / _________ (dd/ mm /yyyy)	99 Unknown
	Time	______ h______	99 Unknown

36) *Gestational age at delivery:* 1 <28 weeks 2 ≥28 weeks at ______ /40 99 Unknown

37) *Did labour occur:* 1 Yes 2 No 99 Unknown

38) *Was labour induced:* (circle all that apply)

1 Artificial rupture of membranes	3 Prostaglandins	5 Oxytocin
2 Mechanical	4 Misoprostol	99 Unknown

39) *Was labour augmented:* (tick all that apply)
1 No 2 Yes 3 Medication used ________________________________
99 Unknown

40) *Was Labour Care Guide used:*
1 Yes, used correctly 2 Yes, incomplete 3 No, not used 4 Not in labour 99 Unknown

41) *Please indicate duration:* (where known)

1	Latent phase (<5cm)	_____/_____ hours / mins	99 Unknown
2	Active phase (5-10cm)	_____/_____ hours / mins	99 Unknown
3	2nd stage of labour (10cm to delivery)	_____/_____ hours / mins	99 Unknown
4	3rd stage of labour (delivery of baby to delivery of placenta)	_____/_____ hours / mins	99 Unknown

42) *Final Mode of Delivery:*

First Baby
- 1 Vaginal Delivery - Cephalic / Breech (circle one)
- 2 Instrumental Delivery – Ventouse / Forceps
- 3 Caesarean section (a) Planned (b) Emergency
  Indication for C/S ________________________
- 4 Laparotomy (Uterine Rupture)
- 5 Other (Specify) __________________________
- 66 Undelivered
- 99 Unknown

Twin 2 Choose number (from above) __________ 99 Unknown

Triplet 3 Choose number (from above) __________ 99 Unknown

55 Miscarriage / Termination (circle one) ______/40

43) *Were there any Pregnancy or Labour Complications* (circle all that apply)*:*

- 1 Premature ROM
- 2 Prolonged ROM
- 3 Premature labour
- 4 Placenta praevia
- 5 Placenta abruptio
- 6 Antepartum haemorrhage
- 7 Hypertension (no preeclampsia)
- 8 Preeclampsia / Eclampsia
- 9 Diabetes / Gestational DM
- 10 Prolonged labour
- 11 Obstructed labour
- 12 Malpresentation
- 13 Shoulder dystocia
- 14 Postpartum haemorrhage
- 15 Uterine rupture
- 16 Cord Prolapse
- 17 Maternal infection/s
- 18 Oligohydramnios
- 19 Polyhydramnios
- 20 Fetal distress
- 21 Meconium stained liquor
- 22 Ectopic / abdominal pregnancy
- 23 Septic miscarriage
- 24 Other (Specify) ______________________
- 25 None
- 99 Unknown

44) *Interventions around the time of Delivery and/or Maternal Death:* (circle all that apply)

Early Pregnancy	1	ERPOC / MVA	6	Hysterotomy
	2	Laparotomy	7	Hysterectomy
	3	Laparoscopy	8	Blood Transfusion
	4	Myomectomy		
	5	Salpingectomy	99	Unknown
Antenatal	1	Blood Transfusion	2	Tocolysis
			99	Unknown
Intrapartum	1	Induction of Labour	4	Caesarean Section
	2	Augmentation of Labour	5	Blood Transfusion
	3	Instrumental Delivery	99	Unknown
Postpartum	1a	Oxytocin	4	Manual removal of placenta
	1b	Tranexamic acid	5	Balloon Tamponade
	1c	Ergometrine	6	Laparotomy
	1d	Misoprostol	7	B-Lynch suture
	1e	Carbetocin	8	Hysterectomy
	2a	Surgical repair of perineal trauma	9	Blood Transfusion/s
	2b	Surgical repair of cervical trauma	10	Other __________________
	3	ERPOC	99	Unknown
Other	1	Local Anaesthesia	6	$MgSO_4$ (AN / Intra / Post partum)
	2	Epidural	7	Invasive Monitoring
	3	General Anaesthesia	8	Ventilation / CPAP
	4	Spinal Anaesthesia	9	Other __________________
	5	Conscious Sedation	99	Unknown

45) *Did complications require above interventions?* 1 Yes _____ 2 No_____

46) *Did above intervention result in complication?* 1 Yes _____ 2 No_____

47) *Summarize the interventions and/or complications resulting from interventions*

____
____
____
____
____

48) *Treatment (include drugs/dosage, surgery etc):* (relevant to cause of death and contributing morbidity)

____
____
____
____
____

E. Pregnancy outcome*

(*skip this section when pregnancy outcome is miscarriage or termination)

49) *Pregnancy outcome:* (circle one - if more than triplets, please add details at bottom of page)

First Baby   1 Live Birth   2 Stillbirth: Antepartum / Intrapartum   99 Unknown
3 Baby alive on admission? Yes / No   99 Unknown

Twin 2   Choose number 1-3 (from above) _______   99 Unknown

Triplet 3   Choose number 1-3 (from above) _______   99 Unknown

50) *Apgar Scores:* (for stillbirths indicate O and O / for miscarriage and termination, skip to Section F)

First Baby	_____ 1min;	_____ 5 min;	_____ 10 min	99 Unknown
Twin 2	_____ 1min;	_____ 5 min;	_____ 10 min	99 Unknown
Triplet 3	_____ 1min;	_____ 5 min;	_____ 10 min	99 Unknown

51) *Sex & Birth measurements:*

First Baby	Sex: M / F	__________ grams	Length _______ cm	HC _______ cm	99 Unknown
Twin 2	Sex: M / F	__________ grams	Length _______ cm	HC _______ cm	99 Unknown
Triplet 3	Sex: M / F	__________ grams	Length _______ cm	HC _______ cm	99 Unknown

F. Maternal Death Details

52) *Death details:* (for stillbirths indicate data and time of Birth)

1 Date   ______ /_____ / ________ (dd/ mm /yyyy)   99 Unknown
2 Time   ______ h_____   99 Unknown

53) *Timing of maternal death:*

1 Early Pregnancy (<20 weeks)
2 Late Pregnancy (>20 weeks)
3 Intrapartum (during labour or within 12 hours of delivery)
4 Postpartum (>12hrs - 72 hrs after delivery)
5 Postpartum (>72 hrs to 42 days after delivery)
99 Unknown (but during pregnancy or <42days)

54) *Place where death occurred:*

1 Before arrival
2 At arrival / during triage
3 After admission: ANW/ Labour /PNW
4 After admission: ICU / ACU
5 After admission: Gynae ward
6 Other ____________________

55) *Manner of Death*

1 Disease
2 Assault
4 Accident
5 Legal intervention
6 War
7 Pending investigation
88 Could not be determined
99 Unknown / Unclear

PLEASE NOTE: WHO (2016) clearly differentiates between the following:

Cause of death:	the disease, injury or complication directly *RESULTING* in death (ultimate consequence)
Underlying cause:	the disease or injury *INITIATING* the sequence of events leading to death or fatal injury
Manner of death:	the circumstances in which a death occurred
Mode of death:	comprises conditions such as heart failure, cardiac arrest, shock, brain failure, hepatic failure, renal failure – these generally do NOT occur without initiating or precipitating causes or illnesses

56) *Maternal death classification:*
Use the table below (WHO, ICD 10MM) to determine the underlying cause of death by choosing the Type, Group and underlying cause of death (select only the most appropriate underlying cause)

Type	Group	Underlying Cause (select only ONE)
Maternal Death: DIRECT	A. Pregnancies with abortive outcomes	1) Septic miscarriage<br>2) Haemorrhage (non-trauma)<br>3) Uterine trauma<br>4) Gestational trophoblastic disease (GTD)<br>5) Following legal TOP<br>6) Ectopic < 20 weeks<br>7) Ectopic > 20 weeks
	B. Hypertensive Disorders	8) Chronic hypertension<br>9) Pre-eclampsia<br>10) Eclampsia<br>11) HELLP<br>12) Liver rupture
	C. Obstetric Haemorrhage	13) Abruption with hypertension<br>14) Abruption without hypertension<br>15) Placenta praevia<br>16) Other APH<br>17) Ruptured uterus with previous CS<br>18) Ruptured uterus without previous CS<br>19) Retained placenta<br>20) Morbidly adherent placenta<br>21) Uterine atony<br>22) Vaginal trauma<br>23) Cervical trauma<br>24) Bleeding during CS<br>25) Bleeding after CS<br>26) Other PPH
	D. Pregnancy related infection	27) Chorioamnionitis (ruptured membranes)<br>28) Chorioamnionitis (intact membranes)<br>29) Puerperal sepsis after vaginal delivery<br>30) Puerperal sepsis after CS<br>31) Bowel trauma at CS<br>32) Bladder trauma
Maternal Death: DIRECT	E. Other obstetric complications	33) Amniotic fluid embolism<br>34) Pulmonary embolism<br>35) Hyperemesis gravidarum<br>36) Acute fatty liver<br>37) Maternal suicide
	F. Unanticipated complications of management	38) Anaesthetic complications (general)<br>39) Anaesthetic complications (spinal)<br>40) Drug related complications<br>a) Antibiotics (e.g. Penicillin)<br>b) $MgSO_4$

Type	Group	Underlying Cause (select only ONE)
		c) Opioids<br>d) Insulin<br>e) ARV medication<br>f) TB medication<br>g) Herbal medication<br>h) Other medication<br>41) Blood transfusion reaction
Maternal Death:<br>INDIRECT	G. Non-obstetric complications	42) Cardiac disease<br>43) Endocrine conditions<br>44) Gastrointestinal tract conditions<br>45) Central nervous system conditions<br>46) Respiratory conditions<br>47) Genitourinary conditions<br>48) Renal disease<br>49) Liver disease<br>50) Autoimmune disorders<br>51) Skeletal diseases<br>52) Psychiatric disorders<br>53) Neoplasms<br>54) Infections that are not a direct result of pregnancy<br>a) PCP pneumonia<br>b) Other Pneumonia<br>c) TB<br>d) Endocarditis<br>e) UTI<br>f) Malaria<br>g) Cryptococcal meningitis<br>h) Other meningitis<br>i) Toxoplasmosis<br>j) Hepatitis<br>k) Gastroenteritis<br>l) Wasting syndrome<br>m) Other<br>n) Disease outbreak or related complications (e.g. Cholera, Covid-19 etc)
Maternal Death:<br>UNSPECIFIED	H. Unknown /<br>I. Undetermined	55) Death at home or outside health services<br>56) No cause found<br>57) Incomplete history / lack of information<br>58) Missing file / records unavailable
Maternal Death:<br>During pregnancy, childbirth and puerperium	J. Coincidental Cause	59) Motor Vehicle Accident<br>60) Other accidents (e.g. drowning, falling etc.)<br>61) Assault<br>99) Other

57) *Cause of Death: (sequence of events leading up to death)*

Immediate CAUSE/ disease/ injury/ complication *directly* leading to death on line (a) — *a)* ______________________

secondary to (as a consequence of) (b) below

Disease/injury/complication leading to (a) above — *b)* ______________________

secondary to (as a consequence of) (c) below

Disease/injury/complication leading to (b) above — *c)* ______________________

Other contributing morbidity ______________________

G. Modifiable Factors

Use the table below to indicate all applicable avoidable factors. Where "Yes" is ticked, provide further details

58) *Woman /Family / Community related modfiable factors:*

Factor	No	Yes	Un known	Details (if yes)
1. No Antenatal Care				
2. Infrequent Antenatal Care				
3. Delay in seeking care				
4. Declined treatment or admission				
5. Unsafe termination of pregnancy				
6. Use of herbal oxytocics				
7. Use of other traditional medicine/s				
8. No healthcare access due to Disease outbreak / Extreme weather / Conflict				

59) *Health System related (Administrative) modfiable factors:*

Factor	No	Yes	Un known	Details (if yes)
9. Lack of transport (home to facility)				
10. Lack of transport (between facilities)				
11. Lack of accessibility				
12. Delay in initiating critical care (overburdened facility)				
13. Communication breakdown (between healthcare workers)				
14. Lack of facilities, equipment or consumables Specify:				
15. Lack of human resources (health care workers, incl. ambulance personnel)				
16. Lack of expertise, training or education				
17. Lack of Specialist				
18. Service interruption due to Disease outbreak / Extreme weather / Conflict				
19. Other				

60) *Health Worker related modifable factors:*

Factor	No	Yes	Un known	Details (if yes)
20. Incorrect / Poor documentation of care				
21. Labour care guide not used or incomplete				
22. Delay in initiating critical care (failure to act promptly)				
23. Delay in referring patient				
24. Managed at inappropriate level				
25. Problem with recognition / diagnosis				
26. Incorrect diagnoses (resulting in incorrect management)				
27. Treatment not provided				

Factor	No	Yes	Un known	Details (if yes)
28. Incorrect treatment or management (but correct diagnosis)				
29. Sub-standard management (but correct diagnosis)				
30. Not monitored / infrequently monitored				
31. Prolonged abnormal monitoring but no action taken				
32. Inappropriate discharge				
33. Other				

61) *Was an Autopsy performed:*

1 Yes (if yes, specify results) 2 No 99 Unknown

____

____

____

H. Case Summary

Please summarize important information, findings and actions relating to the case

____

____

____

____

____

____

____

____

____

____

____

____

I. Conclusion

Would the outcome have changed with improved care?

1. NO, good care was provided
2. NO, improvements to care may have made NO difference to outcome
3. YES, (preventable) improvements to care may have made a difference to outcome

Form completed by:

Full Name ______________________________ Rank ____________

Cell phone ______________________________

email ______________________________

Signature ______________________________ Date ____________
